# Total Alkaloid Extract of Nelumbinis Plumula Promoted Sleep in PCPA-Induced Insomnia Rats by Affecting Neurotransmitters and Their Receptor Activities

**DOI:** 10.3390/ijms26083684

**Published:** 2025-04-13

**Authors:** Wenjun Wei, Dongge Wang, Hangying Li, Hongyu Tian, Zhilei Wang, Suxiang Feng

**Affiliations:** 1 Academy of Chinese Medical Sciences, Henan University of Chinese Medicine, Zhengzhou 450046, China; donggewang01@163.com (D.W.); 13938402379@163.com (H.T.); 13721626283@163.com (Z.W.); 2 Collaborative Innovation Center for Chinese Medicine and Respiratory Diseases Co-Constructed by Henan Province & Education Ministry of China, Zhengzhou 450046, China; 3 Henan Key Laboratory of Chinese Medicine for Respiratory Disease, Zhengzhou 450046, China; 4 College of Pharmacy, Ningxia Medical University, Yinchuan 750004, China; lihy17@lzu.edu.cn

**Keywords:** Nelumbinis Plumula, total alkaloid, insomnia, neurotransmitter

## Abstract

Insomnia seriously affects people’s health and daily life. There is a growing interest in sleep-promoting agents from natural sources. Nelumbinis Plumula (NP), a traditional Chinese medicine with dual food-medicine homology, has the effects of clearing the heart and calming the mind, showing promising efficacy in treating insomnia. In this study, the effects of NP extract, total alkaloid extract of NP, and crude polysaccharide of NP were measured in para-chlorophenylalanine-induced insomnia rats combined with the pentobarbital sodium experiment. The results indicated both total alkaloid extract and NP total extract could improve insomnia in rats, with the total alkaloid extract demonstrating a stronger effect than NP total extract. Total alkaloid extract significantly prolonged sleep duration and shortened sleep latency. Therefore, total alkaloids in NP appeared to be the main pharmacological substances that exerted sedative effect. Simultaneously, total alkaloid extract could increase the GABA level and reduce the DA level as well as affect the activities of GABRA1, DRD2, 5-HT1A, and AChE proteins. This study can lay an experimental foundation for the further development and application of NP as a remedy for treating insomnia.

## 1. Introduction

Insomnia refers to a type of disorder in which patients are unable to obtain sufficient sleep time and depth. A survey report shows up to 35% of the global population suffers from insomnia [1,2]. Chronic insufficient sleep will increase the risks of hypertension, heart disease, diabetes, depression, anxiety, and other diseases, which can seriously affect physical and mental health, as well as daily life of people [1,2,3,4,5]. In clinical practice, synthetic sedative-hypnotics such as benzodiazepines are mainly used to treat insomnia. However, long-term use of these drugs can lead to tolerance and dependence, and abrupt discontinuation can also cause withdrawal symptoms [2,6]. In addition, some antidepressant and anti-anxiety drugs with hypnotic effects are also used in clinical practice to treat insomnia, though these drugs are not developed specifically for insomnia [6,7]. Traditional Chinese medicine (TCM) has a long history of use and plays an important role in treating insomnia, with great potential for further development [8,9,10,11]. Consequently, there is a growing interest in sleep-promoting medicine from natural sources that may potentially present fewer and less severe side effects.

Nelumbinis Plumula (NP), a traditional Chinese medicine used as both food and medicine, is the dried young leaves and embryonic roots of the mature seeds of *Nelumbo nucifera* Gaertn., belonging to Nymphaeaceae family. NP is first recorded in Chen Shiliang’s book “Edible Materia Medica” during the Tang Dynasty, with a long history of medicinal use. Ongoing investigation on NP has revealed its chemical compositions and corresponding pharmacological effects. Phytochemistry investigations have shown that the main chemical components of NP consist of alkaloids, flavonoids, sterols, volatile oils, polysaccharides, etc., among which the total alkaloid components account for approximately 2.0% in the NP. Isoquinoline alkaloids are the most important type of skeleton, with representative alkaloid components including neferine, liensinine, isoliensinine, and nuciferine [12,13,14,15,16,17]. Recent studies indicated that NP essential oil, analyzed via GC-MS, contained multiple bioactive compounds, with antioxidant capacity positively correlated to phenolic and terpenoid content [15]. NP total flavonoids significantly suppressed LPS-induced inflammatory responses in RAW264.7 cells by reducing nitric oxide, prostaglandin E2, cytokines, ROS levels, and (Tumor necrosis factor-alpha) NF-κB p65 nuclear translocation, thereby inhibiting the NF-κB signaling pathway [16]. The isoquinoline alkaloids from NP were as potent inhibitors of melanogenesis through mechanisms involving tyrosinase suppression and modulation of melanogenic signaling pathways, demonstrating their potential as natural depigmenting agents [17]. Additionally, Chen et al. comprehensively reviewed the biological activities of NP, including lowering blood pressure, anti-arrhythmic, antioxidant, anti-platelet aggregation, neuroprotective effects, and so on [14].

As a nervine tonic in TCM practice, NP exhibits cardiotonic and anxiolytic effects, which can be clinically applied for restlessness and insomnia [18]. Jo et al. discovered that NP extract can reduce sleep latency time and increase sleep duration time in a pentobarbital induced sleep model. Simultaneously, the total sleep time and non-rapid-eye-movement (NREM) sleep are significantly prolonged compared with the control group, while the wake-up time and rapid-eye-movement (REM) are significantly shortened [19]. In addition, lotus heart tea is also a kind of Tonic Diet for alleviating the restlessness caused by the accumulation of heart fire in people’s daily lives. However, there are relatively few research reports on the pharmacological substance basis and mechanism of action of NP extract in treating insomnia. The specific bioactive compounds and mechanisms underlying NP’s sleep-promoting effects remain unresolved, hindering clinical standardization.

In this study, the effects of NP extract, total alkaloid extract of NP (NPA), and crude polysaccharide of NP (NPP) were measured in para-chlorophenylalanine (PCPA)-induced insomnia rats by the pentobarbital sodium experiment to discover the main medicinal compositions of NP that help calm the nerves. The possible mechanism of action was explored through immunofluorescence staining and Elisa assays. The sleep–wake cycle is a very complex process, involving multiple pathogenic mechanisms. Modern medical research has fully demonstrated that insomnia is associated with various neurotransmitters, such as norepinephrine, acetylcholine (ACh), serotonin (5-HT), histamine, dopamine (DA), orexin, and gamma-aminobutyric acid (GABA) [20]. Meanwhile, the receptors or proteins corresponding to these neurotransmitters, such as Gamma aminobutyric acid type A alpha1 receptor (GABRA1), 5-Hydroxytryptamine 1A receptor (5-HT1A), Dopamine D2 receptor (DRD2), and others, can also influence sleep [21,22]. Therefore, this study examined the effects of these extracts on neurotransmitters 5-HT, DA, GABA, and ACh, as well as 5-HT1A, DRD2, GABRA1, and acetylcholinesterase (AChE) proteins to explore the possible mechanism behind NP’s effects on improving insomnia. The objective of this study is to identify the key bioactive components and elucidate the underlying mechanisms of NP responsible for its sedative-hypnotic effects, so as to provide some references and theoretical basis for NP with clinically application for restlessness and insomnia.

## 2. Results and Discussion

### 2.1. Analysis Results of Nelumbinis Plumula Extract by UPLC-MS/MS

It is well known that traditional Chinese medicine and other plants can synthesize a large number of metabolites with biological functions. Metabolomic analysis has become a pivotal methodology for investigating bioactive components in TCM, particularly for characterizing secondary metabolites that underpin its therapeutic properties. In this study, metabolomics analysis was conducted on the NP extract to acquire some secondary metabolite information, which better elucidated the components in the NP extract and laid the foundation for further exploration of the pharmacological substances of calming the nerves in the subsequent experiments.

**Figure 1:**
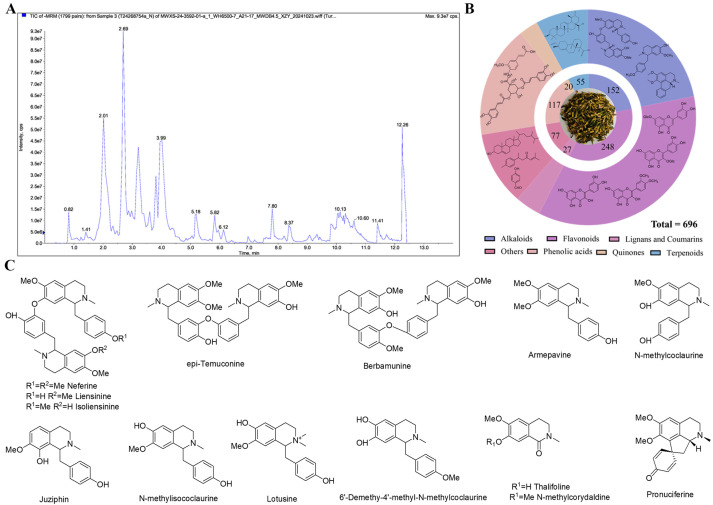
(**A**) Total ion chromatogram of NP extract analyzed by mass spectrometry (Negative ion). (**B**) Statistical results of secondary metabolite types. (**C**) Alkaloids isolated from NP extract.

The UPLC-ESI-QTRAP-MS/MS analysis indicated a total of 696 compounds in the NP extract, as presented in Figure 1A,B, including 248 flavonoids, 152 alkaloids, 117 phenolic acids, 55 terpenoids, 27 lignans and coumarins, 20 quinones, and 77 others. Flavonoids and alkaloids predominated in the extract, with flavones and flavanones as the major flavonoid subclasses and benzylisoquinoline alkaloids representing the primary alkaloid group. Research also evidenced that flavonoids and alkaloids each account for 2% in NP, with a relatively high proportion among all secondary metabolite components [14]. However, the limited number of alkaloids detected (Table S1, Supplementary Materials) likely stems from challenges in isolating alkaloid components. The total alkaloids of NP were also extracted for experimental purposes in this study. At present, we also have isolated some alkaloids from the total alkaloid fraction, as shown in Figure 1C. The pharmacological effects of these alkaloids will also be a focus in our future research.

### 2.2. Animal Experiment and General Condition of Rats

The PCPA-induced insomnia rat is a classic animal model for examining the sedative-hypnotic effect of medicine [8,23,24]. The basic mechanism of this model includes that PCPA can inhibit the production of 5-HT in rats, leading to a significant decrease in the level of 5-HT in the central nervous system of animals and disturbing their circadian rhythm. While injection of PCPA can cause damage to the hippocampal structure, resulting in the changes in the levels of various neurotransmitters [8]. Therefore, the PCPA-induced insomnia rat model was also used in this experiment to explore the effects and mechanisms of various extracts on sleep.

**Figure 2:**
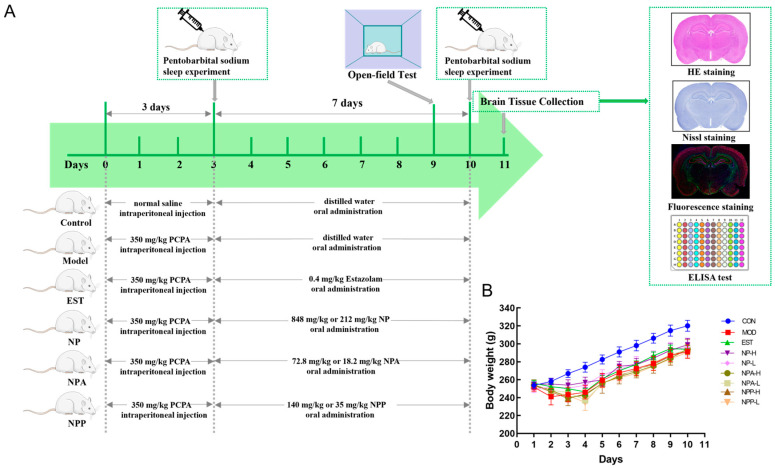
(**A**) Overall conception design of animal experiments and administered dose of NP, NPA, and NPP extracts. (**B**) Changes in body weight of rats in each group (x ± S) (n = 9).

As shown in Figure 2A, 81 male SD rats were randomly assigned into nine groups. During the continuous injection of PCPA, these model rats noticeably displayed heightened daytime activity and restlessness compared with the control group, accompanied by gradual weight loss, which is consistent with established behavioral hallmarks of insomnia. (Figure 2B). On the third day, a pentobarbital sodium experiment was conducted after injecting PCPA for 5 h to verify whether the rat insomnia model was successfully established. Compared with the control group, PCPA-injected rats had longer sleep latency time and shorter sleep duration time. The rat behavioral abnormalities and pentobarbital sodium experiment indicated that the PCPA-induced insomnia model had been successfully established in this study.

Starting from the fourth day, rats in each treated group began to receive oral medication. In our experiment, the total extract, total alkaloid extract, and crude polysaccharide of NP were administered to PCPA-induced insomnia rats by gavage. Given the therapeutic significance of NP for insomnia, this study systematically evaluated these three bioactive fractions to investigate their neuroregulatory effects. Alkaloids were characterized as the dominant small-molecule components with high relative abundance, and polysaccharides was notably abundant in NP.

**Figure 3:**
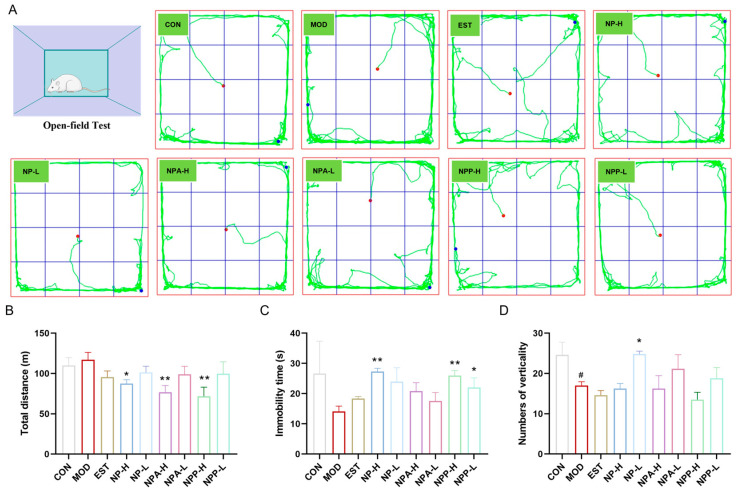
Open field test of rats of each group. (**A**) The movement trajectories of rats of each group (x ± S) (n = 6). (**B**) The total distance traveled by rats of each group (x ± S) (n = 6). (**C**) The immobility of rats of each group. (**D**) The numbers of verticality of rats of each group (x ± S) (n = 6). Compared with the control group, #: *p* < 0.05; compared with the model group, *: *p* < 0.05, **: *p* < 0.01.

After the administration of medication, distinct changes in the behavior of rats were observed on EST, NP-H, NP-L, NPA-H, NPA-L, NPP-H, and NPP-L groups. Compared with the model group, the rats in the treatment groups became docile and had reduced daytime activity. On the ninth day, a behavioral evaluation was performed by an open field test experiment. As shown in Figure 3, compared with the control group, the total movement distance of rats was increased, while the immobility time and the numbers of verticality of rats were decreased in the model group within the same time period. Compared with the model group, the total distance of all treatment group rats showed varying degrees of reduction, especially in the NP-H, NPA-H, and NPP-H groups, which were more visible than the EST group. While the immobility time of all treatment group rats had a significant increase except for the EST group. The numbers of verticality of rats in NP-L, NPA-L, and NPP-L groups were raised compared with the model group, indicating the curiosity and exploratory behavior of rats towards the new environment in these three groups and the blank group.

The behavioral results indicated that NP, NPA, and NPP extracts could reduce anxiety and irritability of rats, allowing them to quickly adapt and maintain a normal state in an unfamiliar environment.

### 2.3. Effects of Various Extracts in PCPA-Induced Insomnia Rats by Pentobarbital Sodium Experiment

**Figure 4:**
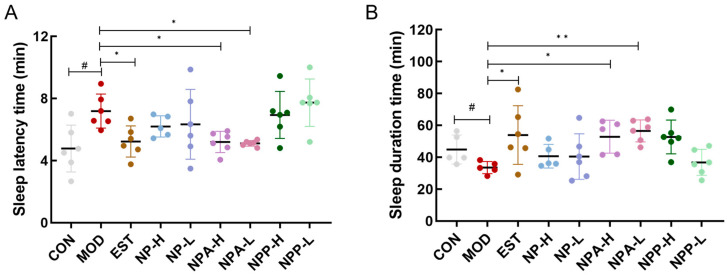
The effects of NP, NPA, and NPP extracts on PCPA-induced insomnia rats. (**A**) Sleep latency time of rats (x ± S) (n = 6). (**B**) Sleep duration time of rats (x ± S) (n = 6). Compared with the control group, #: *p* < 0.05; compared with the model group, *: *p* < 0.05, **: *p* < 0.01.

Subsequently, the pentobarbital sodium experiment was used to investigate the hypnotic and sedative effects of various extracts on PCPA-induced insomnia rats. On the 10th day, all rats were intraperitoneally injected with 35 mg/kg of pentobarbital sodium, and the time of the righting reflex disappearance and the time of waking up from sleep were recorded, which could determine the sleep latency and sleep duration of rats. As presented in Figure 4, compared with the control group, rats in the model group had a significant increase in sleep latency and a noticeable reduction in sleep duration. This result indicated that the injecting PCPA indeed caused insomnia in these rats. Figure 4A displayed that comparing with the model group, the sleep latency of rats in the EST, NPA-H, NPA-L groups was remarkably shortened and reached the same level as that of the control group, with notable differences from the model group. For rats orally administered with NP extract in the NP-H and NP-L groups, NP extract also could slightly cut the sleep latency of those rats. However, the sleep latency of rats in the NPP-H and NPP-L groups was almost the same as that of the model group, indicating that the NP polysaccharide extract could not reduce the sleep latency of PCPA-induced insomnia rats. The statistical results of sleep duration time in each group of rats (Figure 4B) showed that the sleep duration of rats in the EST, NPA-H, NPA-L, and NPP-H groups was increased and had significant differences compared with the model group, which even exceeded that of the control group, particularly in NPA-H and NPA-L groups. Simultaneously, the sleep duration of rats in NP-H and NP-L groups was comparable to that of the control group. However, no visible enhancement in sleep duration of rats was observed in the NPP-L group.

This evidence further illustrated that both NP total alkaloid extract and NP total extract could ameliorate insomnia and exert hypnotic effects in PCPA-induced insomnia rats by shortening sleep latency and prolonging sleep duration. It was particularly emphasized that the hypnotic effect of total alkaloid extract was more pronounced than that of NP extract. Therefore, we hypothesize that NP total alkaloid extract is the primary bioactive compound responsible for sleep-promoting effects in PCPA-induced insomnia rats.

### 2.4. Pathological Observations of HE Staining and Nissl Staining of PCPA-Induced Insomnia Rats

To further explore the impacts of NP, NPA, and NPP extracts on the central nervous system, HE staining and Nissl staining were utilized to observe the neurons in rats across different experimental groups. The rat brain was divided into front and back halves to form a coronal section. In this section, typical structures of the brain such as the complete hippocampus, thalamus, substantia nigra, and hypothalamus could be clearly observed. Through careful comparison, it was observed that PCPA and all extracts had the most pronounced effects on neuronal changes in the hippocampus, as compared with the thalamus, hypothalamus, and substantia nigra. The hippocampus is the most extensively studied part of the cerebral cortex in the central nervous system of mammals, which is associated with learning and memory capacity. Insomnia can lead to severe memory loss, which is closely relevant with the hippocampus. Therefore, this study focused specifically on the neuronal changes in CA1 and CA3 regions of the hippocampal structure of rats.

**Figure 5:**
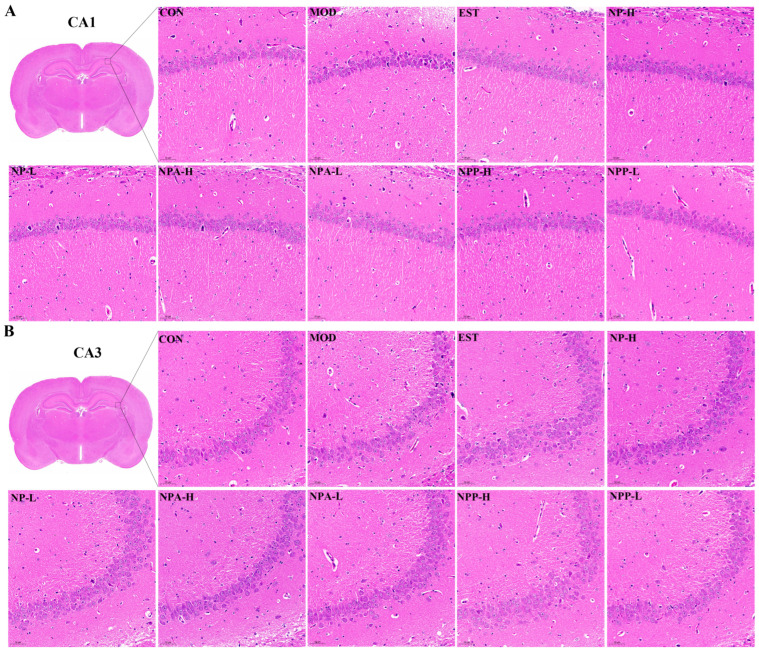
Pathological section of the hippocampus stained by HE in PCPA-induced insomnia rats (n = 3) (magnification × 20, scale bar = 50 μm). (**A**) The effects in the CA1 region of hippocampus. (**B**) The effects in the CA3 region of hippocampus.

HE staining results (Figure 5) displayed that compared with the control group, the neurons of rats were shrunken and decreased, and the boundary between the nucleus and cytoplasm was unclear with cell staining deepening in the model group, indicating that neurons in the hippocampus of the model group rats were damaged to a certain extent owing to intraperitoneal injection of PCPA. Compared with the model group, neuronal damage was less severe in each treatment group, which suggested that NP, NPA, and NPP extracts could alleviate neuronal damage caused by PCPA induced insomnia in different degrees.

**Figure 6:**
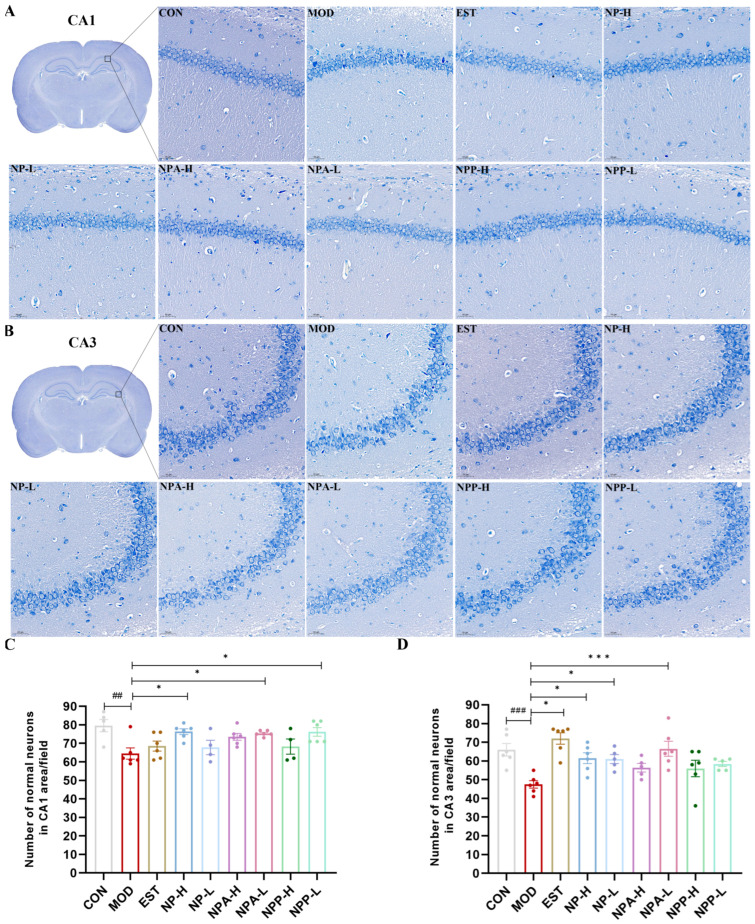
Pathological section of the hippocampus by Nissl staining in PCPA-induced insomnia rats (n = 3) (magnification × 20, scale bar = 50 μm) (**A**) The effects in the CA1 area of hippocampus. (**B**) The effects in the CA3 area of hippocampus. (**C**) Statistics of the number of normal neurons in the CA1 area (x ± S) (n = 6). (**D**) Statistics of the number of normal neurons in the CA3 area (x ± S) (n = 6). Compared with the control group, ##: *p* < 0.01, ###: *p* < 0.001; Compared with the model group, *: *p* < 0.05, ***: *p* < 0.01.

Nissl staining results (Figure 6) showed that the neurons of the CA1 and CA3 regions of the hippocampus in the control group rats were intact, with clear nuclear membrane and abundant Nissl bodies in the cell bodies. In the model group, neurons of rats suffered certain damage, with Nissl bodies decreasing, nuclei shrinking, and the nuclear membrane of some cells disappearing, resulting in the entire cell being deeply stained blue. For the treatment groups, Nissl bodies reappeared and increased in the cytoplasm of neurons during the process of injury recovery. In addition, the number of intact neurons that were not damaged in the CA1 and CA3 regions of the hippocampus was also quantified, as shown in Figure 6C,D. The results showed that compared with the control group, the number of intact and undamaged neurons in the model group was significantly reduced. Compared with the model group, the number of the neurons of NP-H, NPA-H, NPA-L, and NPP-L group rats in the CA1 region increased with significant differences, but the enhancement numbers of neurons in the EST, NP-L, and NPP-H group rats were relatively low. In the CA3 region, the number of neurons evidently increased in the EST, NP-H, NP-L, and NPA-L group rats with significant differences, particularly in the EST and NPA-L groups. However, compared with the model group, there was a slight increase in the numbers of neurons in NPA-H, NPP-L, and NPP-H group rats in the CA3 region.

Overall, the injection of PCPA could cause damage to neurons in the hippocampus of rats, leading to neuronal atrophy and disappearance of the nuclear membrane. All extracts and the positive drug were able to repair neuronal damage of rats and increase the number of normal neurons in the hippocampus to varying degrees.

### 2.5. Determination of Neurotransmitters and Inflammatory Factors in Hippocampus of Rats

The sleep–wake cycle is a very complex process with multiple pathogenic mechanisms. The foundation of the sleep–wake cycle is mainly laid by the neurotransmitters, such as norepinephrine, histamine, orexin, Ach, 5-HT, DA, and GABA [8,20,23]. GABA, a non-proteinogenic amino acid, is the main inhibitory neurotransmitter in the mammalian brain, which is also a known agent for ameliorating sleep disturbances [21,25]. DA, a monoamine catecholamine neurotransmitter, exerts excitatory effects by activating receptors in the central nervous system, thereby propagating neuronal excitation and regulating arousal states [25]. ACh, a cholinergic neurotransmitter, exhibits distinct release patterns across sleep stages: its levels are significantly reduced during non-rapid eye movement (NREM) sleep and sharply elevated during rapid eye movement (REM) sleep, as demonstrated by electrophysiological studies [26]. 5-HT is an important inhibitory monoamine neurotransmitter, playing a crucial role in sleep regulation [8,9,23]. In addition, the changes in pro-inflammatory and anti-inflammatory factors can also affect sleep and wakefulness. Pro-inflammatory cytokines can promote sleep, while anti-inflammatory cytokines tend to attenuate sleep responses induced by sleep promoting stimuli [27]. Among them, pro-inflammatory cytokines interleukin-1 (IL-1) and tumor necrosis factor-alpha (TNF-α) are the two most widely studied in regulating sleep investigation [28]. Hence, in this experiment, the levels of neurotransmitters (GABA, DA, ACh, 5-HT) as well as inflammatory factors (IL-1, TNF-α) in the hippocampus of rats in different group were measured using the Elisa kits.

**Figure 7:**
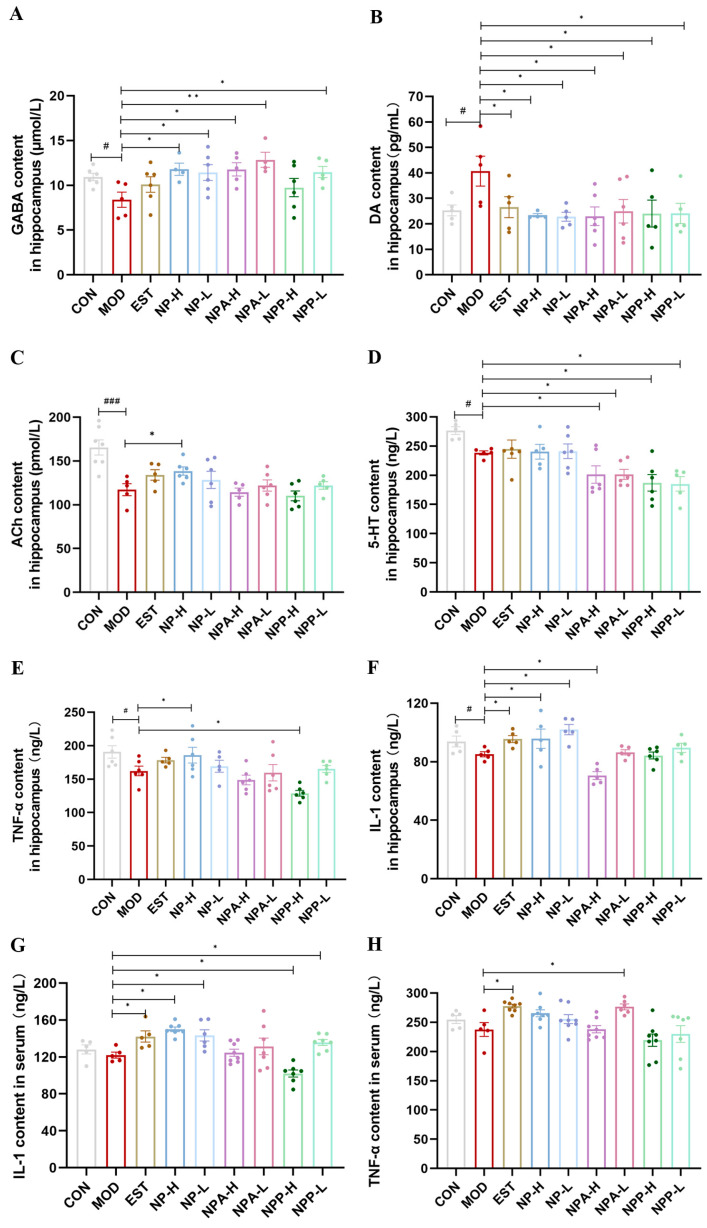
The effects of NP, NPA, and NPP extracts on neurotransmitters and inflammatory factors in hippocampus and serum of PCPA-induced insomnia rats (x ± S) (n = 6). (**A**) GABA levels in hippocampus. (**B**) DA levels in hippocampus. (**C**) ACh levels in hippocampus. (**D**) 5-HT levels in hippocampus. (**E**) TNF-α levels in hippocampus (**F**) IL-1 levels in hippocampus. (**G**) IL-1 levels in serum. (**H**) TNF-α levels in serum. Compared with the control group, #: *p* < 0.05,, ###: *p* < 0.001; Compared with the model group, *: *p* < 0.05, **: *p* < 0.01.

As shown in Figure 7A, the GABA content in the model group was obviously decreased compared with that in the control group, indicating that injection of PCPA in the model group of rats resulted in a reduction in GABA content. Compared with the model group, positive drug and other extracts were able to elevate GABA content to varying degrees in EST, NP, NPA, and NPP groups. Particularly, the NP and NPA extracts had better effects on increasing GABA content than the positive drug. The changes in DA content in the hippocampus of each group rats were shown in Figure 7B. Compared with the control group, the DA content of rats was evidently elevated in the model group, suggesting that the increase in excitatory neurotransmitter DA in the brain of the model group rats contributed to rat insomnia. Compared with the model group, each treatment group could significantly reduce the DA content, reaching a level comparable to that of the control group. As shown in Figure 7C, the ACh content obviously was enhanced in the model group compared with the control group, indicating that the insomnia caused by PCPA affected the release of Ach during REM sleep. Compared with the model group, the Ach levels in the EST, NP-L, and NP-H groups were up-regulated, while the NPA-L and NPP-L extracts could only slightly elevate the content of ACh. Conversely, the NPA-H and NPP-H extracts had few effects on the Ach content. For the 5-HT content in the hippocampus of each animal group, as presented in Figure 7D, its content in the model group was lowered compared with that in the control group, displaying that injection of PCPA could reduce the level of 5-HT. However, compared with the model group, none of the extracts and the positive drug increased the content of 5-HT, indicating that these extracts did not improve insomnia by regulating 5-HT level.

Then, the effects of each extract on inflammatory factors were also assessed. The changes in the content of inflammatory factors TNF-α and IL-1 in hippocampus were presented in Figure 7E,F. Compared with the control group, the contents of TNF-α and IL-1 in the model group were decreased, and the lower contents of TNF-α and IL-1 would affect sleep. In contrast, Estazolam and NP extract were able to improve the contents of TNF-α and IL-1. However, the NPP and NPA groups had opposite effects, reducing the levels of TNF and IL-1. Subsequently, the changes in the levels of IL-1 and TNF-α in the serum were further measured, as shown in Figure 7G,H. The results suggested that their changes and trends were similar to those in the hippocampus. Basically, only the NP extract and Estazolam could significantly affect the IL-1 and TNF-α contents in the serum.

In brief, the insomnia improvement of NP total extract might be closely related to the changes in neurotransmitters (GABA, DA, ACh), as well as pro-inflammatory cytokines (IL-1, TNF-α), with raising the levels of GABA, ACh, IL-1, TNF-α and diminishing the levels of DA. While total alkaloid extract of NP might ameliorate insomnia by increasing DA level and reducing GABA level. Especially, the effects of both total alkaloid and total extract on GABA and DA levels were remarkable with significant differences. Unfortunately, NP, NPA, and NPP extracts cannot affect the 5-HT level to promote sleep.

### 2.6. Results of Immunofluorescence Staining

To further explore the possible protein targets, the modulation effects of NP, NPA, and NPP extracts on the expressions of NeuN, GFAP, GABRA1, DRD2, 5-HT1A, and AChE proteins in the hippocampus of each group of rats were assessed using immunofluorescence technique. The mean fluorescence intensity of the CA1 and CA3 regions in the hippocampus was, respectively, calculated to quantify the effects of various extracts on the expression of related proteins.

**Figure 8:**
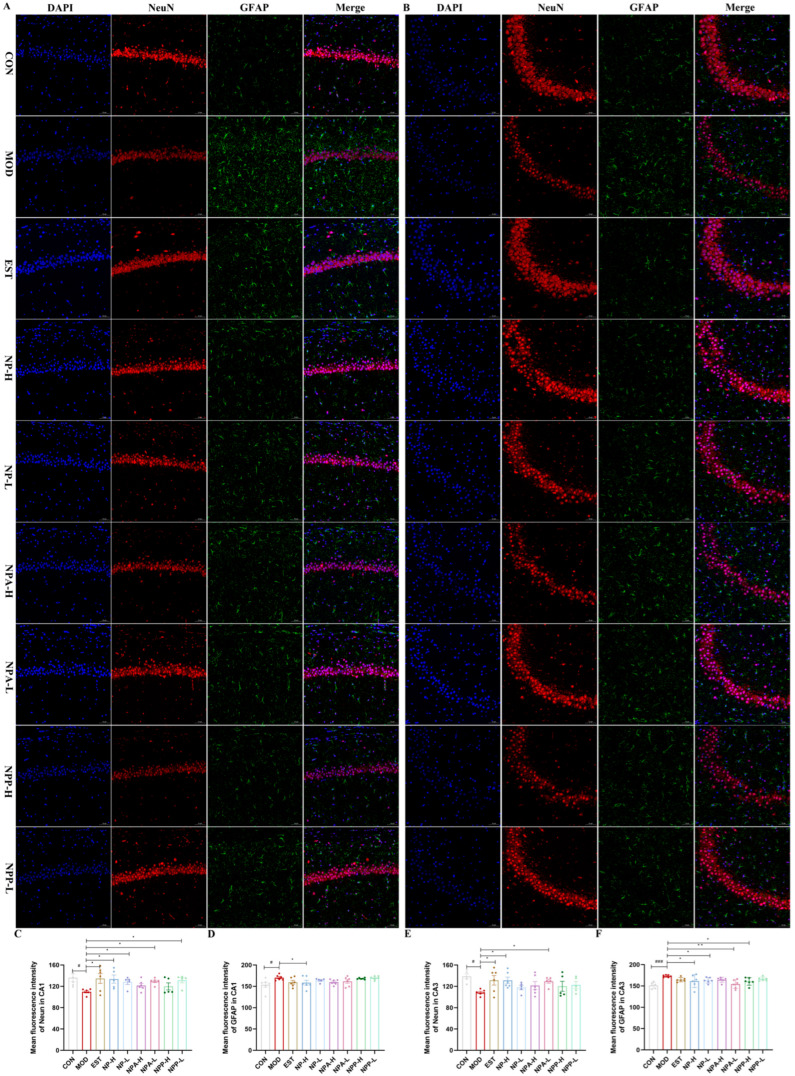
The effects of NP, NPA, and NPP extracts on NeuN and GFAPs in hippocampus of PCPA-induced insomnia rats (n = 3) (magnification × 20, scale bar = 50 μm). (**A**) The effects in the CA1 area of hippocampus. (**B**) The effects in the CA3 area of hippocampus. (**C**) Quantification of the protein expression of NeuN in the CA1 area (x ± S) (n = 6). (**D**) Quantification of the protein expression of GFAP in the CA1 area (x ± S) (n = 6). (**E**) Quantification of the protein expression of NeuN in the CA3 area (x ± S) (n = 6). (**F**) Quantification of the protein expression of GFAP in the CA3 area (x ± S) (n = 6). Compared with the control group, #: *p* < 0.05, ###: *p* < 0.001; Compared with the model group, *: *p* < 0.05, **: *p* < 0.01.

Neuronal nuclei (NeuN), as a biomarker, is specifically expressed in neurons, which is detected exclusively in post-mitotic neurons. NeuN is initially identified through an immunological screen to produce neuron-specific antibodies [29]. The changes in NeuN expression are related to neuronal degeneration, with the highest NeuN immunoreactivity mainly occurring in neurons in all cortical and hippocampal pyramidal layers [30]. Glial fibrillary acidic protein (GFAP) provides structural support and maintains the mechanical integrity of astrocytes. As a biomarker protein for the activation of astrocytes, GFAP may exert a variety of physiological effects in neurological diseases. When the central nervous system is damaged by chemicals, the expression of GFAP will increase [31]. Therefore, elevated GFAP is a hallmark signal of the nervous system’s response to injury. As shown in Figure 8, in both CA1 and CA3 regions, the mean fluorescence intensity of NeuN in the model group was significantly lower than that in the control group, while its fluorescence intensity of each treatment group was higher than that in the model group. Especially in the NP-H and NPA-L groups, their mean fluorescence intensity was comparable to that of the positive drug. The fluorescence intensity of GFAP in the model group was significantly higher than that in the control group, while all treatment groups were able to reduce the content of GFAP compared with the model group, but the effect was less significant. The above results indicated that injection of PCPA caused a certain degree of damage for the neurons of rats, and each NP extract could repair the damage with protective effects.

GABRA1, also known as GABA type A receptor subunit alpha 1, is a ligand-gated chloride ion channel that primarily functions in the central nervous system. The mechanism of action of GABRA1 is to regulate the opening of chloride ion channels by binding to GABA, leading to hyperpolarization of neuronal cell membrane and thereby reducing neuronal excitability. GABRA1 participates in regulating the excitability of the nervous system, which is crucial for maintaining its normal function in biological significance. Benzodiazepines, widely used in insomnia treatment, act as positive allosteric modulators at the GABRA1 of ionotropic GABAA receptors, potentiating chloride ion influx and neuronal hyperpolarization. Therefore, the activation of GABRA1 is beneficial for sleep induction, positioning GABAA receptors as a primary target for seeking natural anxiolytic compounds or sedatives [8,21,32,33].

**Figure 9:**
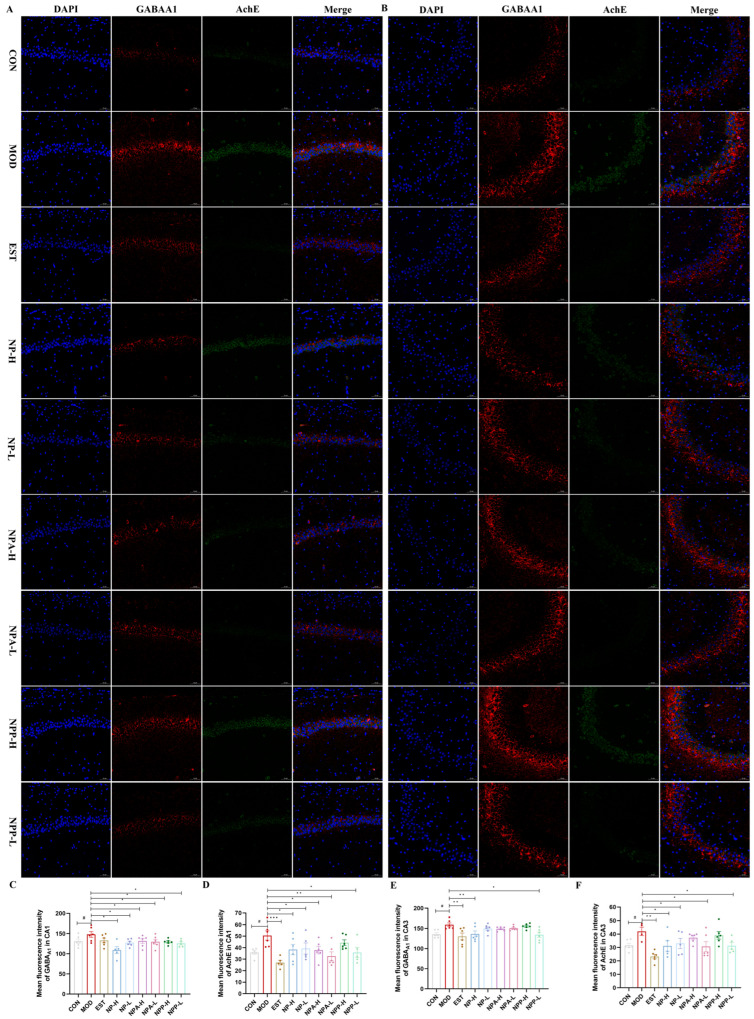
The effects of NP, NPA, and NPP extracts on GABRA1 and AChE proteins in hippocampus of PCPA-induced insomnia rats (n = 3) (magnification × 20, scale bar = 50 μm). (**A**) The effects in the CA1 area of hippocampus. (**B**) The effects in the CA3 area of hippocampus. (**C**) Quantification of the protein expression of GABRA1 in the CA1 area (x ± S) (n = 6). (**D**) Quantification of the protein expression of AChE in the CA1 area (x ± S) (n = 6). (**E**) Quantification of the protein expression of GABRA1 in the CA3 area (x ± S) (n = 6). (**F**) Quantification of the protein expression of AChE in the CA3 area (x ± S) (n = 6). Compared with the control group, #: *p* < 0.05; Compared with the model group, *: *p* < 0.05, **: *p* < 0.01, ***: *p* < 0.001.

AChE is a key enzyme in biological nerve conduction, which can degrade acetylcholine and terminate the excitatory effect of neurotransmitters on the postsynaptic membrane. GABRA1 is involved in cell development and maturation and can promote neuronal development and regeneration [26]. Hence, the effects of NP, NPA, and NPP extracts on the GABRA1 and AChE proteins were also examined in this experiment. In the CA1 region (Figure 8A,C,D), the expression levels of GABRA1 and AChE proteins in the model group were significantly higher than those in the control group, especially AchE expression with significant differences. After administration, the expression levels of GABRA1 in all groups decreased compared with that in the model group, of which the expression levels of GABRA1 in the NPA and NPP groups were comparable to that of the positive group and reached the same level as that of the control group, while the effect of the NP extract was stronger than that of the positive group. As for the expression level of the AChE protein in the CA1 region, all groups showed a very significant reduction compared with the model group, except for NPP-H group, with the NPA-L and EST groups displaying more obvious effects than other groups. As presented in Figure 9B,E,F, the statistical analysis of the mean fluorescence intensity in the CA3 region revealed that all treatment groups were able to reduce the expression of GABRA1 and AChE proteins except for the NPP-H group. For the expression of GABRA1, the effects of NP-H and NPP-L groups were comparable to that of the EST group, reaching the same level as the control group. The expression of AChE in the NPA-L and NPP-L groups was the same as that in the control group. Therefore, each treatment group had more significant effects on the expressions of GABRA1 and AChE proteins in the CA1 area than in the CA3 area. In particular, NP extract can significantly affect the GABRA1 and AChE protein expressions, while total alkaloid extract prominently affects the protein expression of AChE.

**Figure 10:**
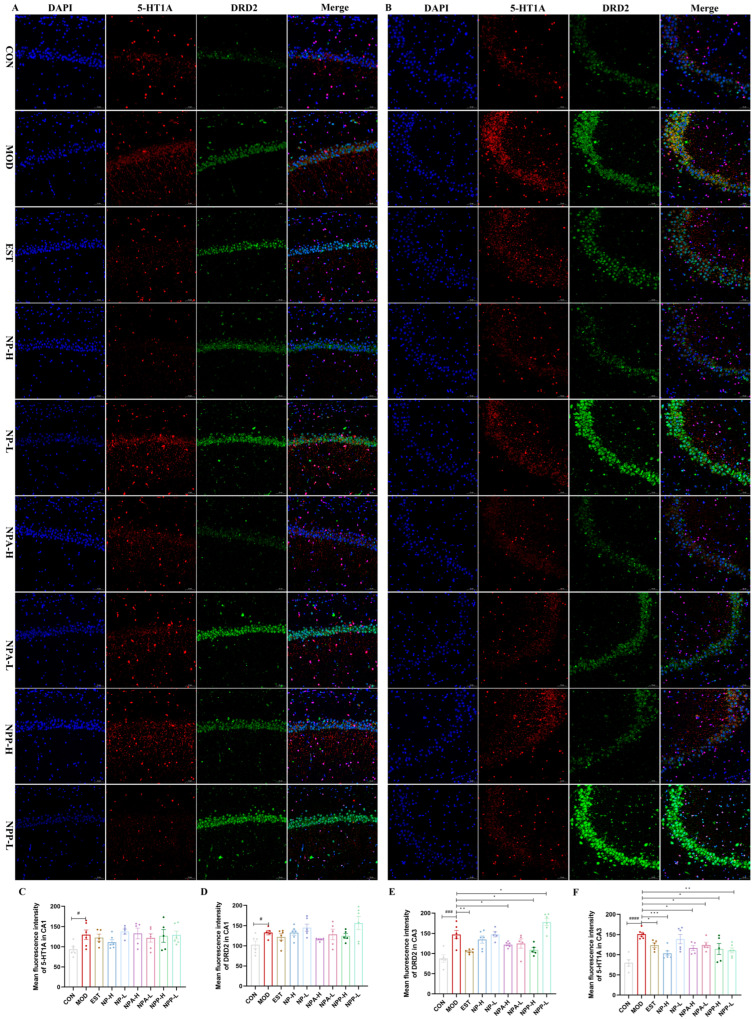
The effects of NP, NPA, and NPP extracts on 5-HT1A and DRD2 proteins in hippocampus of PCPA-induced insomnia rats (n = 3) (magnification × 20, scale bar = 50 μm). (**A**) The effects in the CA1 area of hippocampus. (**B**) The effects in the CA3 area of hippocampus. (**C**) Quantification of the protein expression of 5-HT1A in the CA1 area (x ± S) (n = 6). (**D**) Quantification of the protein expression of DRD2 in the CA1 area (x ± S) (n = 6). (**E**) Quantification of the protein expression of DRD2 in the CA3 area (x ± S) (n = 6). (**F**) Quantification of the protein expression of 5-HT1A in the CA3 area (x ± S) (n = 6). Compared with the control group, #: *p* < 0.05, ###: *p* < 0.001, ####: *p* < 0.0001; compared with the model group, *: *p* < 0.05, **: *p* < 0.01, ***: *p* < 0.001.

The 5-HT receptors simultaneously regulate excitatory and inhibitory neurotransmitters in the brain, including glutamate, norepinephrine, GABA, DA, and ACh. Among the 5-HT receptor subtypes, the 5-HT1A receptor plays a pivotal role in sleep–wake cycle regulation by modulating serotonergic neurotransmission [8,21,22]. Dopamine receptors are divided into D1 and D2 types, with D2 type receptors having a much greater affinity for endogenous DA than D1 type receptors. DRD2 plays a crucial role in maintaining wakefulness. Previous investigations have shown that knocking out DRD2 in animals results in a significant reduction in wakefulness [23,34]. In this experiment, the effects of NP, NPA, and NPP extracts on the 5-HT1A and DRD2 proteins were also measured by immunofluorescence technique. In the CA1 region of hippocampus shown in Figure 10A,C,D, the expression levels of DRD2 and 5-HT1A in the model group were higher than those in the control group, indicating that an injection of PCPA would cause overexpression of these two proteins. Compared with the model group, the positive group, NP-H group, and NPA-L group were able to reduce 5-HT1A expression, with the NP-H group having a more significant effect than the positive group, while other groups showed little change. Compared with the model group, the NPA-H, NPA-L, and NPP-H groups were able to reduce the expression level of DRD2, and the reduction effect was more notable than that of the positive group. Similarly, compared with the model group, the expression levels of DRD2 in the CA3 region could be reduced in all groups, except for the NPP-L group. And for the expression levels of 5-HT1A, all groups could also decrease its expression with significant differences. In comparison, the NP, NPA, and NPP extracts showed more remarkable expression for these two proteins in the CA3 region. In short, NP-H extract can significantly affect the 5-HT1A protein expression, while the total alkaloid extract prominently affects the protein expression of DRD2.

Therefore, NP and NPA extracts could improve PCPA induced insomnia, which were related to activities of GABRA1, DRD2, 5-HT1A, and AChE proteins in the hippocampus. The effect of NP extract on GABRA1 in both the CA1 and the CA3 regions was relatively remarkable. NP extract displayed a stronger effect on AChE expression in the CA1 region than in the CA3 region. NP extract had a significant effect on the 5-HT1A protein in the CA1 region and a relatively small effect on the CA3 region, with little effect on the expression of DRD2 in the CA1 and the CA3 regions. For NPA extract, its effect on GABRA1 in the CA1 region was stronger than that in the CA3 region, and it had a more prominent effect on AchE in the CA1 region. The effect of NPA extract on DRD2 expression in the CA3 and CA1 regions was significant, while it had a relatively low impact on 5-HT1A expression in the CA1 region and a certain effect in the CA3 region. In brief, NP total alkaloid extract can significantly affect DRD2 and AChE protein expressions, while NP total extract prominently affects GABRA1 and AChE protein expressions.

In summary, the UPLC-ESI-QTRAP-MS/MS analysis of NP total extract indicated that flavonoids and alkaloids accounted a relatively high proportion among all secondary metabolite components, with alkaloids being characteristic compositions in NP, especially isoquinoline alkaloids with higher content. In the PCPA-induced insomnia rat experiment, both NP total alkaloid extract and NP total extract were effective in improving insomnia in rats, and total alkaloid extract displayed a stronger effect than NP extract, significantly prolonging sleep duration time and shortening sleep latency time. Therefore, total alkaloids may be the main pharmacological substances that exert sedative effects in NP.

Regarding the mechanisms of the NP alkaloid in improving insomnia, this study focuses on neurotransmitter systems modulating the sleep–wake cycle, particularly GABAergic and dopaminergic pathways. GABA, the primary inhibitory neurotransmitter in the mammalian central nervous system, promotes sleep induction through postsynaptic GABAA receptor-mediated chloride influx. Conversely, DA, an excitatory catecholamine, maintains wakefulness via receptor activation. Ach release follows a biphasic pattern, decreasing during NREM sleep and surging during REM sleep. 5-HT modulates sleep architecture through dual mechanisms: 5-HT1A receptors in the dorsal raphe nucleus inhibit serotonin release during sleep onset, while 5-HT2C receptors in the hypothalamus promote wakefulness via orexin co-activation. Thus, fluctuations in these indicators evidently affect animal sleep behavior.

The insomnia improvement of NP total extract may be closely related to the levels of neurotransmitters GABA, DA, and Ach, as well as pro-inflammatory cytokines IL-1 and TNF-α. The effect of NP total alkaloid extract on promoting sleep may be associated with increasing DA and reducing GABA levels. It was worth mentioning that the effects of both total alkaloid and total extract on GABA and DA levels were remarkable with significant differences. Additionally, both NP total extract and NP total alkaloid extract may promote sleep by affecting the activities of GABRA1, DRD2, 5-HT1A, and AChE proteins. Especially, NP total alkaloid extract can significantly affect DRD2 and AChE protein expressions, while NP total extract prominently affects GABRA1 and AChE protein expressions. It is speculated that the impacts of these two extracts on sleep may be closely related to DAergic and GABAergic systems, suggesting that NP total extract and NP total alkaloid extract ameliorated insomnia through multiple pathways and targets.

Currently, total alkaloids might be the primary pharmacological substance of NP for alleviating insomnia. We have already isolated several alkaloid components from the NP total alkaloid extract. Further studies are warranted to determine which alkaloid constituents in NP have the effects on sleep-related behaviors.

## 3. Materials and Methods

### 3.1. Plant Materials

Nelumbinis Plumula, produced in Shangrao, Jiangxi province, was purchased in September 2023. The Nelumbinis Plumula medicinal herb had been identified by Professor Suxiang Feng from Henan University of Chinese Medicine, which was stored in Henan University of Chinese Medicine (voucher specimen: NP20230901).

### 3.2. Sample Preparation

#### 3.2.1. NP Extract Preparation

Nelumbinis Plumula medicinal material of 50 kg was refluxed and extracted twice with 80% ethanol for 2 h. The extracted solution was concentrated under reduced pressure to obtain 10.6 kg of the crude extract, with an extraction yield of 21.1%.

#### 3.2.2. NPA Preparation

The crude extract was suspended in an aqueous solution, and 2% sulfuric acid aqueous solution also was added to adjust the pH to 2–3. Then, the acidic mixed solution was extracted with ethyl acetate twice. After extraction, ammonia was added to the aqueous solution to adjust the pH to 9–10. Finally, alkaline solution was extracted three times with dichloromethane to obtain the total alkaloid extract (NPA), with an extraction yield of 1.82%.

#### 3.2.3. NPP Preparation

Nelumbinis Plumula was crushed and extracted with 95% ethanol reflux three times, and the remaining Nelumbinis Plumula powder was dried at 40 °C in an oven. Then, drying Nelumbinis Plumula powder was added into distilled water for ultrasonic extraction three times. After filtering the extracted solution, four times the volume of anhydrous ethanol was added and then the mixed solution was left to stand at 4 °C for 12 h. After settling, the polysaccharide precipitate was separated from the solution using a centrifuge. The separated polysaccharide precipitate was dissolved again with distilled water to remove protein using the Sevag method for a pure polysaccharide solution. Finally, the Nelumbinis Plumula polysaccharide (NPP) was obtained by freeze-drying, with an extraction yield of 3.5%.

### 3.3. Reagents

GABA (MM-0441R1), DA (MM-0355R1), 5-HT (MM-0442R1), ACh (MM-0195R1), IL-1 (MM-0046R1), and TNF-α (MM-0180R1) Elisa kits were bought from Jiangsu Meimian Industrial Co., Ltd. (Yancheng, Jiangsu, China). The GABRA1 (14471-1-AP) and DRD2 (55084-1-AP) primary antibodies were purchased from Proteintech Group, Inc. (Wuhan, Hubei, China). NeuN (GB11138), GFAP (GB15096), 5-HT1A (GB114285), and AChE (GB12011) primary antibodies were bought from Servicebio Technology Co., Ltd. (Wuhan, China). Hematoxylin and Eosin (HE) staining kit and Nissl dye solution were purchased from Servicebio Technology Co., Ltd. (Wuhan, Hubei, China). PCPA was purchased from Sigma-Aldrich (St. Louis, MO, USA). Estazolam tablets with lot No. H37023047 were used as the positive control drug and manufactured by Shandong Xinyi Pharmaceutical Co., Ltd. (Dezhou, Shandong, China). Pentobarbital sodium with lot No. H31021725 for rat anesthesia and sedation hypnosis was purchased from Shanghai Shangyao Xinya Pharmaceutical Co., Ltd. (Shanghai, China).

### 3.4. Analysis of Secondary Metabolite by UPLC-MS/MS

The SHIMADZU Nexera X2 UPLC system coupled to Applied Biosystems 4500 QTRAP Tandem mass spectrometer (Agilent Technologies Inc., Santa Clara, CA, USA) was employed to analyze the NP extract. The secondary metabolite qualitative analysis was carried out according to secondary spectral information based on the Metware Database (Wuhan Maiwei Metabolic Biotechnology Co., Ltd., Wuhan, China). The UPLC conditions were as follows: the chromatographic column was an Agilent SB-C18 (2.1 mm × 100 mm, AB SCIEX, Foster City, CA, USA); the mobile phase conditions consisted of A: water (0.1% formic acid) and B: acetonitrile (0.1%formic acid); the elution gradient program was: 0–9 min, 95:5 to 5:95, 9–10 min, 5:95, 10–11 min, 5:95 to 95:5, 11–14 min, 95:5, volume ratio of A to B; the flow rate was 0.35 mL/min; the column temperature box was maintained at 40 °C; the injection volume was 2 μL. The mass spectrometry conditions were optimized as follows: the electrospray ion source (ESI) temperature was set to 500 °C, with an ion spray voltage of 5500 V for positive ion mode and −4500 V for negative ion mode. The ion source gases I (GSI), II (GSII), and curtain gas (CUR) were maintained at 50, 60, and 25 psi, respectively, while the collision-induced ionization parameter was set to high. The QQQ scan utilized multiple reaction monitoring (MRM) mode with medium nitrogen as the collision gas. The declustering potential (DP) and collision energy (CE) for each MRM ion pair were optimized further. A specific set of MRM ion pairs was monitored during each period according to the eluted metabolites.

### 3.5. Animal Administration

SPF-grade SD male rats (170–200 g, 7 weeks old) were purchased from Beijing Vitonglihua Experimental Animal Technology Co., Ltd. (Beijing, China), all of which were used to establish a PCPA-induced insomnia rat model. The production license number of laboratory animals is SCXK (Beijing) 2021-0006 with an animal quality certificate (No.110011241104238381). The laboratory animal ethical review was approved by the Scientific Ethics Committee of Center for Laboratory Animals in Henan University of Chinese Medicine (Approval No. IACUC-202405015). All rats were housed in an environment with a constant temperature of 25 ± 1 °C, a relative humidity of 60 ± 10%, and a 12 h light-dark cycle in Animal Experiment Center of Henan University of Chinese Medicine. All rats were acclimated and fed in the same environment for 7 days before the experiment officially began.

### 3.6. PCPA-Induced Insomnia Animal Model

After seven days of adaptive feeding, 81 SPF-grade SD male rats were randomly divided into nine groups with nine rats in each group, consisting of the control group (CON, blank control), model group (MOD), Estazolam group (EST, positive control), high-dose NP extract group (NP-H), low-dose NP extract group (NP-L), high-dose NP total alkaloid extract group (NPA-H), low-dose NP total alkaloid extract group (NPA-L), high-dose NP crude polysaccharide group (NPP-H), and low-dose NP crude polysaccharide group (NPP-L). Nine rats in the control group and the remaining 72 rats in the other groups were intraperitoneally injected with normal saline and 350 mg/kg/d PCPA for three consecutive days, respectively. The method for establishing this PCPA-induced insomnia rat model was described in the literature [8,23,24]. Then, the various extracts and positive drug were administered by gavage from the fourth day for seven consecutive days, once every day. Rats in the EST group were administered Estazolam (0.4 mg/kg). Rats in NP-H and NP-L groups were given 848 mg/kg and 212 mg/kg of the NP crude extract, respectively. Rats in the NPA-H and NPA-L groups were given 72.8 mg/kg and 18.2 mg/kg of the NP total alkaloid extract, respectively. Rats in NPP-H and NPP-L groups were given 140 mg/kg and 35 mg/kg of the NP crude polysaccharide, respectively. A total of 18 rats in the CON and MOD groups were administered the same volume of distilled water for seven consecutive days. On the 9th day, an open-field test was conducted to record the trajectories of all rats. The result of behavioral experiment was analyzed using a rat behavior analysis system (XinSoft Information Technology Co., Ltd., Shanghai, China).

### 3.7. Pentobarbital Sodium Sleep Experiment

After injecting PCPA five hours on the 3rd day and the 10th day, all rats were given intraperitoneally with subthreshold dose of pentobarbital sodium (35 mg/kg, 0.1 mL/10 g), as referenced in the literature [8,24]. Then, the time of rats with the disappearance of righting reflex was recorded, as well as the sleep duration. The purpose of conducting this experiment on the third day was to verify whether the insomnia animal model was successful.

### 3.8. HE Staining

On the 10th day, the rats were euthanized, and their brains and other organs were quickly and carefully separated on an ice tray. Then, the whole brains of 27 rats (3 rats per group) were fixed in 4% paraformaldehyde for embedding and sectioning in the following experiments. Then, these brain sections were sequentially treated with hematoxylin and eosin (H&E). After dehydration and sealing, the stained sections were observed under a vertical optical microscope (NIKON ECLIPSE E100, Tokyo, Japan) and then scanned.

### 3.9. Nissl Staining

The whole brains of 27 rats (3 rats per group) were fixed in 4% paraformaldehyde to perform the Nissl staining. These brain tissues were embedded and sectioned. Next, the environmentally friendly dewaxing solution and gradient ethanol (100%–75%) were utilized to dewax and dehydrate the sections, respectively. Subsequently, the sections were treated with a Nissl staining solution and immersed in clean xylene for 10 min and mounted. Finally, stained sections were observed in a microscope (NIKON ECLIPSE E100, Japan), and the hippocampal neurons were quantified using Fiji Image J software (Version Fiji 1.53t WIN, NIH, Bethesda, MD, USA).

### 3.10. Enzyme-Linked Immunosorbent Assay

On the 10th day, the rats were euthanized, and their brains and other organs were quickly and carefully separated on an ice tray. Then, hippocampal tissues were isolated from the remaining 6 brains in each group of rats and processed using a homogenizer to obtain homogenates. The above homogenates were further centrifuged at 4 °C to acquire the supernatant. The contents of 5-HT, GABA, DA, ACh, IL-1, and TNF-α were measured using commercial ELISA kits according to the manufacturers’ protocols.

### 3.11. Immunofluorescence Staining

The whole brains of 27 rats (3 rats per group) were fixed in 4% paraformaldehyde for fluorescence homologous double staining experiment. Brain sections were successively dewaxed and dehydrated by the environmentally friendly dewaxing solution and ethanol, as described in the reference [5,35]. After completing antigen repair, sections were placed in PBS (pH 7.4) to wash on a decolorization shaker. Sections were put in a 3% hydrogen peroxide solution and incubated at room temperature in the dark for 25 min to block endogenous peroxidase. Then, sections were washed three times in PBS (pH 7.4) on a decolorization shaker. The PBS was shaken off, bovine serum albumin was added dropwise and sealed for 30 min. Next, the sections were, respectively, incubated overnight at 4 °C with the following primary antibodies: NeuN 1:200/GFAP 1:500, DRD2 1:500/5-HT1A 1:5000, and GABRA1 1:300/AChE 1:500, following that the fluorescent secondary antibodies (CY3 labeled goat anti-rabbit IgG for GABRA1 and NeuN, Alexa Fluor 488 labeling goat anti-mouse IgG for E and GFAP, as well as HRP labeled goat anti-rabbit IgG for DRD2 and 5-HT1A) were incubated for 50 min at room temperature. After adding DAPI staining solution dropwise, the sections were incubated at room temperature for 10 min in the dark environment. After sealing with anti-fluorescence quenching sealing agent, images were collected using a fluorescence microscope (Nikon Eclipse C1, Yokohama, Japan).

### 3.12. Statistical Analysis

Statistical analysis was processed by using SPSS software (Version 26.0, IBM, Armonk, NY, USA). The experiment results were presented in the form of mean ± standard error (SE). All data were evaluated by one-way ANOVA and *p* < 0.05 was considered statistically significant.

## 4. Conclusions

This study establishes Nelumbinis Plumula total alkaloids (NPA) as the principal bioactive constituents responsible for its sedative-hypnotic effects, surpassing the crude extract in PCPA-induced insomnia models. Mechanistically, NPA modulates sleep–wake homeostasis through dual regulation of DAergic and GABAergic systems, evidenced by its ability to elevate DA levels and reduce GABA levels, which represents a novel mechanism distinct from single-target synthetic hypnotics, highlighting NPA’s potential as a multi-target therapeutic.

Future research should focus on isolating bioactive alkaloids to verify their respective roles in sleep regulation and search for active lead compounds, which must be followed by comprehensive in vivo pharmacokinetic profiling to systematically delineate the bioavailability, blood–brain barrier permeability, and metabolic stability of NP alkaloids. The following research will provide valuable references for the development of alkaloid new drugs in the future.

## Data Availability

The data presented in this study are available on request from the corresponding author.

## Supplementary Materials

The following supporting information can be downloaded at: https://www.mdpi.com/article/10.3390/ijms26083684/s1.

## Abbreviations

The following abbreviations are used in this manuscript:

TCM	Traditional Chinese medicine
PCPA	DL-4-chlorophenylalanine
GABA	Gamma-aminobutyric acid
DA	Dopamine
ACh	Acetylcholine
5-HT	5-Hydroxytryptamine
IL-1	Interleukin-1
TNF-α	Tumor necrosis factor-alpha
GABRA1	Gamma aminobutyric acid type A alpha1 receptor
5-HT1A	5-Hydroxytryptamine 1A receptor
DRD2	Dopamine D2 receptor
AChE	Acetylcholinesterase
NREM	Non-rapid-eye-movement
REM	Rapid-eye-movement
NPA	Nelumbinis Plumula total alkaloid
NP	Nelumbinis Plumula
NPP	Nelumbinis Plumula crude polysaccharide
NeuN	Neuronal nuclei antigen
GFAP	Glial fibrillary acidic protein
HE	Hematoxylin and Eosin staining
UPLC	Ultra high performance liquid chromatogram
ELISA	Enzyme-linked immunosorbent assay
