# Supplementary material for: Total Alkaloid Extract of Nelumbinis Plumula Promoted Sleep in PCPA-Induced Insomnia Rats by Affecting Neurotransmitters and Their Receptor Activities
Source: Int J Mol Sci. 2025 Apr 13;26(8):3684. doi: 10.3390/ijms26083684 (PMC12027186; doi:10.3390/ijms26083684)
Supplement: Supplementary file 1 [file ijms-26-03684-s001.zip › ijms-3545523-supplementary.pdf]

Supporting information

Table S1. Alkaloids in NP extract obtained through metabolomics analysis

Compounds	Types	Formula	Q1 (Da)	Q3 (Da)
Neferine	Isoquinoline	C38H44N2O6	625.33	206.12
Liensinine	Isoquinoline	C37H42N2O6	609.3	297.14
Isoliensinine	Isoquinoline	C37H42N2O6	609.3	403.18
Norlotusine	Isoquinoline	C18H22NO3+	300.1582	107.05
Nor-O-methylarmepavine	Isoquinoline	C19H23NO3	314.18	107.05
(S)-Norcoclaurine*	Isoquinoline	C16H17NO3	272.13	107.05
Norisoliensinine*	Isoquinoline	C36H40N2O6	597.29	192.11
Didesmethylneferine*	Isoquinoline	C36H40N2O6	597.29	192.11
Lotusine	Isoquinoline	C19H23NO3	314.18	299.15
Thalifoline	Isoquinoline	C11H13NO3	208.1	151.08
Jatrorrhizine	Isoquinoline	C20H20NO4+	338.14	323.12
Pseudopalmatine	Isoquinoline	C21H22NO4+	352.15	336.12
Reticuline-glucose	Isoquinoline	C25H33NO9	492.22	330.17
Norjuziphine	Isoquinoline	C17H19NO3	286.14	107.05
Magnocurarine	Isoquinoline	C19H24NO3+	314.17	269.12
Tetrahydroprotopapaverine*	Isoquinoline	C19H23NO4	330.17	192.1
Salsolidine	Isoquinoline	C12H17NO2	208.13	160.09
Stepharine	Isoquinoline	C18H19NO3	298.14	192.1
Dauricine	Isoquinoline	C38H44N2O6	625.33	206.12
Oblongine	Isoquinoline	C19H24NO3+	314.17	269.12
Demethylarmepavine Glucoside*	Isoquinoline	C24H31NO8	462.21	300.16
Norarmepavine Glucoside	Isoquinoline	C24H31NO8	462.21	300.16
Hydrohydrastinine*	Isoquinoline	C11H13NO2	192.1	177.08
(S)-Coclaurine*	Isoquinoline	C17H19NO3	286.14	107.05
N-Methylhigenamine*	Isoquinoline	C17H19NO3	286.14	107.05
(1s)-1-[(4-hydroxyphenyl)methyl]-7-methoxy-1,2,3,4-tetrahydroisoquinolin-8-ol*	Isoquinoline	C17H19NO3	286.14	107.05
1-[(4-methoxyphenyl)methyl]-1,2,3,4-tetrahydroisoquinoline-6,7-diol*	Isoquinoline	C17H19NO3	286.14	107.05
Argemexirine*	Isoquinoline	C17H19NO3	286.1441	107.05
1-[(4-hydroxyphenyl)methyl]-7-methoxy-1,2,3,4-tetrahydroisoquinolin-8-ol*	Isoquinoline	C17H19NO3	286.14	107.05
1-(4-hydroxyphenyl)-7-methoxy-1,2,3,4-tetrahydroisoquinolin-8-ol*	Isoquinoline	C16H17NO3	272.13	107.05
(1r)-1-(4-hydroxyphenyl)-7-methoxy-1,2,3,4-tetrahydroisoquinolin-8-ol*	Isoquinoline	C16H17NO3	272.13	107.05
(1r)-1-[(4-hydroxyphenyl)methyl]-7-methoxy-2-methyl-3,4-dihydro-1h-isoquinolin-8-ol	Isoquinoline	C18H21NO3	300.16	107.05

Dehydroyanhunine*	Isoquinoline	C21H22NO4+	352.16	337.13
Yanhusuine*	Isoquinoline	C21H22NO4+	352.16	337.13
13,13α-Didehydro-9,10-dimethoxy-2,3-(methylenedioxy)-berbine*	Isoquinoline	C20H19NO4	338.14	322.11
3-[2-(dimethylamino)ethyl]-6-methoxy-2-[(1e)-2-(4-methoxyphenyl)ethenyl]phenol	Isoquinoline	C20H25NO3	328.19	253.09
(1r)-1-[(4-methoxyphenyl)methyl]-1,2,3,4-tetrahydroisoquinoline-6,7-diol*	Isoquinoline	C17H19NO3	286.14	107.05
Thebaine	Isoquinoline	C19H21NO3	312.16	296.13
Thebainone	Isoquinoline	C18H21NO3	300.16	164.07
13-Methylpalmatrubine	Isoquinoline	C21H22NO4+	352.15	308.13
4'-Hydroxy-6,7-dimethoxyl-N,N-dimethyltetrahydroisoquinoline	Isoquinoline	C20H25NO3	328.19	313.13
N-methylhigenamine-7-O-glucopyranoside*	Isoquinoline	C23H29NO8	448.2	286.14
norcoclaurine-6-O-glucoside*	Isoquinoline	C22H27NO8	434.18	255.11
13-Methyldehydrocorydalmine*	Isoquinoline	C21H22NO4+	352.15	337.13
Palmatine*	Isoquinoline	C21H22NO4+	352.15	337.13
3-Hydroxy-N-methylcoclaurine Glucoside*	Isoquinoline	C24H31NO9	478.21	316.15
5'-Hydroxy-N-methylcoclaurine Glucoside*	Isoquinoline	C24H31NO9	478.21	316.15
Glaziovine	Isoquinoline	C18H19NO3	298.14	283.12
Berbamunine	Isoquinoline	C36H40O6N2	597.3	192.1
8-Oxyberberine	Isoquinoline	C20H17NO5	352.12	337.1
Epiberberine	Isoquinoline	C20H18NO4+	336.12	320.09
Corydayanine	Isoquinoline	C20H20NO4+	338.14	322.11
Nornuciferine	Aporphine	C18H19NO2	282.1489	265.1224
Sinomendine*	Aporphine	C20H19NO4	338.14	323.12
Dehydronuciferine	Aporphine	C19H19NO2	294.15	263.13
Norcorydine	Aporphine	C19H21NO4	328.15	206.08
1,2,9,10-tetramethoxy-6-methyl-6H-dibenzo[de,g]quinoline	Aporphine	C21H21NO4	352.15	336.12
Nuciferine	Aporphine	C19H21NO2	296.16	250.1
N-Acetylanonaine	Aporphine	C19H17NO3	308.12	249.09
(-)-Annonaine	Aporphine	C17H15NO2	266.12	191.09
O-Nornuciferine (N-Methylasimilobine)(Floribundine)	Aporphine	C18H19NO2	282.15	236.08
(+)-Pronuciferine	Aporphine	C19H21NO3	312.16	269.12
N-Nornuciferine	Aporphine	C18H19NO2	282.15	250.11
Aporheine	Aporphine	C18H17NO2	280.13	249.1
Roemerine	Aporphine	C18H17NO2	280.13	249.1
3-amino-2-naphthoic acid*	Alkaloids	C11H9NO2	188.07	118.06
naphthisoxazol A*	Alkaloids	C11H9NO2	188.07	118.06

4-Methylazetidine-2-Carboxylic acid*	Alkaloids	C5H9NO2	116.07	70.06
N-methylcoclaurine-glucose*	Alkaloids	C24H31NO8	462.21	300.16
Dihydroberberine*	Alkaloids	C20H19NO4	338.14	323.12
Tetraphyllin A*	Alkaloids	C12H17NO6	272.11	110.06
N-[7'-(4'-Methoxyphenyl)ethyl]-2-methoxybenzamide*	Alkaloids	C17H19NO3	286.14	107.05
Normorphine*	Alkaloids	C16H17NO3	272.13	107.05
Higenamine-glucose	Alkaloids	C22H27NO8	434.18	272.13
N-(2-Hydroxy-4-methoxyphenyl)acetamide	Alkaloids	C9H11NO3	182.08	91.05
Heterodendrin	Alkaloids	C11H19NO6	262.13	216.12
Tyramine	Alkaloids	C8H11NO	138.09	103.05
Hexadecanamide	Alkaloids	C16H33NO	256.26	88.08
alanine betaine	Alkaloids	C5H11NO2	118.08	72.08
Octadecadienamide	Alkaloids	C18H33NO	280.26	81.07
3-(4-aminobut-1-en-1-yl)-4,7-dihydro-1-indole-5,6-dione	Alkaloids	C12H14N2O2	219.11	176.07
8-[3-(3,4-dihydro-1H-isoquinolin-2-yl)propoxy]-12H-isoindolo[1,2-b]quinazolin-10-one	Alkaloids	C27H25N3O2	424.21	160.08
3-(2-amino-1-hydroxyethyl)-1H-indol-5-ol	Alkaloids	C10H12N2O2	193.1	148.04
N-benzoyl-2-aminoethyl-β-D-glucopyranoside	Alkaloids	C15H21NO7	328.13	310.12
10-Hydroxymethyllycaconitine	Alkaloids	C37H50N2O11	699.35	699.35
6,7-dimethoxy-2-methyl-1,2-dihydroisoquinoline	Alkaloids	C12H15NO2	206.12	190.08
demethylcoclaurine-glucose*	Alkaloids	C22H27NO8	434.18	255.1
4-(2-aminoethenyl)phenol*	Alkaloids	C8H9NO	136.07	91.05
Styrylamine	Alkaloids	C8H9N	120.08	77.04
Nicotianamine	Alkaloids	C12H21N3O6	304.15	185.09
N-Benzylmethylene isomethylamine	Alkaloids	C8H9N	120.08	103.05
Zarzissine	Alkaloids	C5H5N5	136.06	119.04
5-Methoxynoracronycine*	Alkaloids	C20H19NO4	338.14	322.1
N-benzylformamide*	Alkaloids	C8H9NO	136.07	91.05
coclaurine-glucose*	Alkaloids	C23H29NO8	448.2	286.15
2-Phenylacetamide*	Alkaloids	C8H9NO	136.08	91.05
6-Ethylmorphine	Alkaloids	C19H23NO3	314.17	269.12
Heterocodeine	Alkaloids	C18H21NO3	300.16	237.09
Betaine	Alkaloids	C5H11NO2	118.09	58.07
Daminozide	Alkaloids	C6H12N2O3	161.09	143.08
N-Oleoylethanolamine	Alkaloids	C20H39NO2	326.31	62.06
Argentinine	Alkaloids	C19H21NO2	296.17	191.09
4,5-Dihydropiperlonguminine	Alkaloids	C16H21NO3	276.16	135.04
N-Isobutyl-2E,4E-dodedienamide	Alkaloids	C16H29NO	252.23	196.2

Hordenine-O-α-L-rhamnopyranoside	Alkaloids	C16H25NO5	312.17	121.07
Guineensine	Alkaloids	C24H33NO3	384.25	283.17
Isopiperlonguminine	Alkaloids	C16H19NO3	274.14	201.05
(S)-N-Methylcoclaurine	Alkaloids	C18H21NO3	300.16	107.05
vasicinone	Alkaloids	C11H10N2O2	203.08	130.07
4-Hydroxypipecolic acid	Alkaloids	C6H11NO3	146.08	82.06
6-propargyl-5,6,6a,7-tetrahydro-1-hydroxy-2,9,10-trimethoxy-4H-dibenzo(de,g)quinoline	Alkaloids	C22H23NO4	366.17	350.14
6,7-dimethoxy-1-methylisoquinoline	Alkaloids	C12H13NO2	204.1	188.07
Aurantiamide acetate	Alkaloids	C27H28N2O4	445.21	224.11
Ajaconine	Alkaloids	C22H33NO3	360.25	360.25
Acetylcholine	Alkaloids	C7H16NO2+	146.12	87.04
Laudanine	Alkaloids	C20H25NO4	344.19	299.13
18-Demethylparaensidimerin C	Alkaloids	C29H28N2O4	469.22	307.13
α-Sanshool	Alkaloids	C16H25NO	248.2	79.06
Erythroculine	Alkaloids	C20H25NO4	344.19	58.07
Guanosine 3'-phosphate	Alkaloids	C10H14N5O8P	362.05	211
2-Glucosyl-glucosyloxy-2-phenylacetic acid amide	Alkaloids	C20H29NO12	474.16	312.1
Armepavine	Alkaloids	C19H23NO3	314.17	58.07
Armepavine-glucose	Alkaloids	C25H33NO8	476.23	314.18
5,6-Dehydrolupanine	Quinorisidine	C15H22N2O	247.18	179.15
Sophocarpine	Quinorisidine	C15H22N2O	247.18	179.15
Lupanine	Quinorisidine	C15H24N2O	249.2	136.11
Caulophylline	Quinorisidine	C12H16N2O	205.13	58.07
3,5-Dihydro-2H-Furo[3,2-C]Quinolin-4-One*	Quinoline	C11H9NO2	188.07	118.07
Dihydroevocarpine	Quinoline	C23H35NO	342.28	173.08
3-hydroxy-1-methylpyrrolidin-2-one*	Pyrrole	C5H9NO2	116.07	70.06
Retronecine	Pyrrole	C8H13NO2	156.1	110.07
3-pyridine-methanol-O-β-D-glucopyranosyl*	Pyridine	C12H17NO6	272.11	110.06
3-Indoleacrylic acid*	Plumerane	C11H9NO2	188.07	118.07
5-Methoxytryptophol*	Plumerane	C11H13NO2	192.1	177.08
N-Hydroxytryptamine	Plumerane	C10H12N2O	177.1	160.08
Indole-3-acetyl-L-aspartic acid	Plumerane	C14H14N2O5	289.08	88.04
2-oxindole-3-acetic acid	Plumerane	C10H9NO3	192.06	146.06
1-(1-aminoethyl)-9a,11a-dimethyl-tetradecahydrocyclopenta[a]phenanthren-7-one	Plumerane	C21H35NO	318.28	219.17
Indoline	Plumerane	C8H9N	120.08	103.05
L-Pipecolate	Piperidine	C6H11NO2	130.09	84.08
1-[1-O-9(3,4-methylenedioxyphenyl)-	Piperidine	C21H27NO3	342.21	229.1

2E,8E-nonadienyl]-piperidine				
1-[1-O-5(3,4-methylenedioxyphenyl)-2E-pentenyl]-piperidine	Piperidine	C17H21NO3	288.16	161.1
2-O-α-D-glucosyl-1-deoxynojirimycin	Piperidine	C12H23NO9	326.15	164.09
Pipecolic acid	Piperidine	C6H11NO2	130.1	56.05
Cinnamoyltyramine*	Phenolamine	C17H17NO2	268.13	131.05
N-(4-hydroxyphenethyl)cinnamamide*	Phenolamine	C17H17NO2	268.13	131.05
N-trans-cinnamoylphydroxyphenylethylamine*	Phenolamine	C17H17NO2	268.13	131.05
Norephedrin 3-O-(2''-Phenylpropanyl)Glucoside	Phenolamine	C24H33NO6	432.24	119.09
Dopamine 4-O-Glucoside	Phenolamine	C14H21NO7	316.14	137.06
N-2-Phenylethyl-cinnamamide	Phenolamine	C17H17NO	252.14	131.05
Feruloylspermidine	Phenolamine	C17H27N3O3	322.21	177.06
Sinapine	Phenolamine	C16H24NO5+	310.16	251.09

Table S2. The retention time, DP and CE of 30 key metabolites

Compounds	Index	RT (min)	DP	CE
Neferine	pmp000467	3.5	40	30
Liensinine	MWSmce687	3.4	-50	-30
Isoliensinine	MWSmce628	3.7	-50	-30
Norlotusine	Jmhp002680	2.49	50	30
Nor-O-methylarmepavine	Jmhp004228	3.6	50	30
(S)-Norcoclaurine*	Cmxp002615	2.6	50	30
Norisoliensinine*	Jmhp003833	3.3	50	30
Didesmethylneferine*	Jmhp003564	3.1	50	30
Lotusine	pmp000463	2.9	40	30
Thalifoline	MWStz189	4.1	50	30
Jatrorrhizine	MWSmce359	4.5	50	30
Pseudopalmatine	Hmdp006927	5	50	30
Reticuline-glucose	Wahlp02439	2.4	50	60
Norjuziphine	Jmhp004382	3.8	50	30
Magnocurarine	Lhbp073003	2.5	80	30
Tetrahydroprotopapaverine*	Cmyp003227	3.2	50	30
Salsolidine	MWSmce316	2.6	50	30
Stepharine	HJAP166	3.1	50	30
Dauricine	MWSslk193	12.2	50	30
Oblongine	Lhbp073004	3.1	80	30
Demethylarmepavine Glucoside*	Wahlp02093	2.1	50	50
Norarmepavine Glucoside	WaJp002408	2.5	50	30
Hydrohydrastinine*	Cmyp001700	2	50	30
(S)-Coclaurine*	Wdhp002681	2.4	50	30
N-Methylhigenamine*	Jmhp002906	2.7	50	30
Nornuciferine	pmp000457	4.35	60	20
Sinomendine*	Lmqp002330	4.1	50	30
Dehydronuciferine	MWSmce599	9.5	50	30
Norcorydine	Hmdp003543	2.7	50	30
Nuciferine	MWSmce223	4.6	50	30
